# The outer diameter of the endoscope is important when performing endoscopic transcanal myringoplasty^☆^

**DOI:** 10.1016/j.bjorl.2017.03.003

**Published:** 2017-03-22

**Authors:** Zhengcai Lou

**Affiliations:** The Affiliated YiWu Hospital of Wenzhou Medical University, Department of Otorhinolaryngology, Zhejiang, China

Dear Editor,

We would like to address the manuscript entitled “Transcanal endoscopic myringoplasty: A case series in a university center” by Garcia et al.^1^ The work is excellent. However, we believe that the appropriate application of the technology, and the surgical indications, are not entirely clear.

The authors write, in the Materials and Methods: “All patients older than 12 years with a diagnosis of non-suppurative, non-cholesteatomatous COM sequela, or with traumatic perforations without spontaneous resolution for more than three months, were included in the study. The diagnosis was based on anamnesis, physical examination, audiometry, and impedance audiometry”.^1^ These inclusion criteria are vague. Previous studies have suggested that most traumatic perforations tend to heal spontaneously within 3 months of injury.^2^ Certain simple, noninvasive treatments facilitate the closure of subacute tympanic membrane perforations; these include topical FGF-2 application^3^ and Gelfoam patching. Thus, endoscopic cartilage myringoplasty simply increases medical costs, and complications, when traumatic perforations do not undergo spontaneous resolution within 3 months. In addition, the authors do not make it clear whether perforations with otitis media of the type associated with granulation tissue were included. Endoscopic myringoplasty alone is not reliable in such patients; mastoidectomy should also be performed.

The authors write, in the Materials and Methods: “The technique and the surgical instruments used were the same as in routine surgeries with microscope, except for the non-use of the microscope andotologic speculum, and the use of the Storz rigid endo-scopes, 4 mm diameter and 18 cm long, at 0° angulation (Karl Storz GmbH & Co. KG-Tuttlingen, Germany)”.^1^ The authors do not report the status of the External Auditory Canal (EAC). We have found that endoscopic myringoplasty is particularly difficult in patients with tortuous or extremely narrow EACs, and in those with any mass (such as a small osteoma) that reduces the endoscopic field of view. This is because simultaneous insertion of the endoscope and the requisite surgical instruments crowds the surgical field. Adult EACs of diameter <4.0 mm are considered narrow.^4^ The diameter of the micro-instrument used during otology exceeds 1 mm. The surgical field becomes very crowded if the EAC is <4.0 mm in diameter, particularly if an endoscope 4.0 mm in outer diameter is employed. However, in children, Ito et al.^5^ believed that endoscopic myringoplasty was feasible when the difference between the diameter of the endoscope and the smallest EAC diameter was >0.5 mm. It was dangerous to employ a 2.7 mm-diameter endoscope during pediatric myringoplasty when the diameter of the EAC was <3.2 mm. Thus, transcanal endoscopic myringoplasty may be difficult in children aged 12–16 years if an endoscope 4.0 mm in outer diameter is used. Furthermore, in most cases, only one hand is free to perform endoscopic myringoplasty because the other must hold the endoscope. A hematoma (with subsequent bleeding) can develop if the endoscope contacts the EAC or the freshened perforation margins of pediatric patients exhibit chronic EAC inflammation, a remnant eardrum, or fungal otitis externa. A massive bleed may obstruct the endoscopic field-of-view, thus hindering surgery. Although application of a sponge soaked in epinephrine (1:100,000 dilution) for a few minutes may afford adequate hemostasis, one-handed surgery using an endoscopic technique prolongs the operation. However, the surgeon can use one hand to aspirate blood and the other to freshen the perforation margins if the bimanual microscopic approach is employed. In addition, the authors write, in the Results section: “As for the surgical outcome at postoperative otoscopy, complete closure of the perforation was observed in 86.4% (*n* = 19) of patients three months after intervention.”^1^ The follow-up time was short and thus the reported success rate is not completely reliable. Most authors suggest that follow-up for at least 1 year is necessary; re-perforation after myringoplasty is possible during this time.^6^

In brief, the transcanal endoscopic approach is excellent for performing cartilage myringoplasty, reducing both the surgical time and complications. However, both the surgical indications, and careful consideration of the outer diameter of the endoscope, are very important, and long-term follow-up is required.

## Conflicts of interest

The author declares no conflicts of interest.

## References

[1] Garcia Lde B., Moussalem G.F., Andrade J.S., Mangussi-Gomes J., Cruz O.L., Penido Nde O. (2016). Transcanal endoscopic myringoplasty: a case series in a university center. Braz J Otorhinolaryngol.

[2] Jellinge M.E., Kristensen S., Larsen K. (2015). Spontaneous closure of traumatic tympanic membrane perforations: observational study. J Laryngol Otol.

[3] Lou Z., Huang P., Yang J., Xiao J., Chang J. (2016). Direct application of bFGF without edge trimming on human subacute tympanic membrane perforation. Am J Otolaryngol.

[4] Cole R.R., Jahrsdoerfer R.A. (1990). The risk of cholesteatoma in congenital aural stenosis. Laryngoscope.

[5] Ito T., Kubota T., Watanabe T., Futai K., Furukawa T., Kakehata S. (2015). Transcanal endoscopic ear surgery for pediatric population with a narrow external auditory canal. Int J Pediatr Otorhinolaryngol.

[6] Mohamad S.H., Khan I., Hussain S.S. (2012). Is cartilage tympanoplasty more effective than fascia tympanoplasty. A systematic review. Otol Neurotol.

